# Post-discharge changes in nutritional status predict prognosis in patients with acute decompensated HFpEF from the PURSUIT-HFpEF Registry

**DOI:** 10.1007/s00380-024-02499-y

**Published:** 2024-12-10

**Authors:** Takashi Kitao, Shungo Hikoso, Shunsuke Tamaki, Masahiro Seo, Masamichi Yano, Takaharu Hayashi, Akito Nakagawa, Yusuke Nakagawa, Yohei Sotomi, Daisaku Nakatani, Takahisa Yamada, Yoshio Yasumura, Yasushi Sakata, the OCVC-Heart Failure Investigators, Masahiro Seo, Tetsuya Watanabe, Takahisa Yamada, Takaharu Hayashi, Yoshiharu Higuchi, Masaharu Masuda, Mitsutoshi Asai, Toshiaki Mano, Hisakazu Fuji, Daisaku Masuda, Shunsuke Tamaki, Ryu Shutta, Shizuya Yamashita, Masami Sairyo, Yusuke Nakagawa, Haruhiko Abe, Yasunori Ueda, Yasushi Matsumura, Kunihiko Nagai, Masamichi Yano, Masami Nishino, Jun Tanouchi, Yoh Arita, Nobuyuki Ogasawara, Takamaru Ishizu, Minoru Ichikawa, Yuzuru Takano, Eisai Rin, Yukinori Shinoda, Koichi Tachibana, Shiro Hoshida, Masahiro Izumi, Hiroyoshi Yamamoto, Hiroyasu Kato, Kazuhiro Nakatani, Yuji Yasuga, Mayu Nishio, Keiji Hirooka, Takahiro Yoshimura, Yoshinori Yasuoka, Akihiro Tani, Yasushi Okumoto, Yasunaka Makino, Toshinari Onishi, Katsuomi Iwakura, Yoshiyuki Kijima, Takashi Kitao, Hideyuki Kanai, Masashi Fujita, Koichiro Harada, Masahiro Kumada, Osamu Nakagawa, Ryo Araki, Takayuki Yamada, Akito Nakagawa, Yoshio Yasumura, Taiki Sato, Akihiro Sunaga, Bolrathanak Oeun, Hirota Kida, Yohei Sotomi, Tomoharu Dohi, Kei Nakamoto, Katsuki Okada, Fusako Sera, Hidetaka Kioka, Tomohito Ohtani, Toshihiro Takeda, Daisaku Nakatani, Hiroya Mizuno, Shungo Hikoso, Yasushi Sakata

**Affiliations:** 1 Department of Cardiology, Minoh City Hospital, 5-7-1 Kayano, Minoh, Osaka 562-0014 Japan; 2 Department of Cardiovascular Medicine, Osaka University Graduate School of Medicine, 2-2 Yamadaoka, Suita, 565-0871 Japan; 3 Department of Cardiology, Rinku General Medical Center, 2-23 Ourai-Kita, Rinku, Izumisano, Osaka 598-8577 Japan; 4 Division of Cardiology, Osaka General Medical Center, 3-1-56 Mandaihigashi, Sumiyoshi-Ku, Osaka, 558-8558 Japan; 5 Division of Cardiology, Osaka Rosai Hospital, 3-1179 Nagasonecho, Kita-Ku, Sakai, 591-8025 Japan; 6 Cardiovascular Division, Osaka Police Hospital, 10-31 Kitayamacho, Tennojiku, Osaka, 543-0035 Japan; 7 Division of Cardiology, Amagasaki Chuo Hospital, 1-12-1 Shioe, Amagasaki, Hyogo 660-0892 Japan; 8 Department of Medical Informatics, Osaka University Graduate School of Medicine, 2-2 Yamadaoka, Suita, 565-0871 Japan; 9 Division of Cardiology, Kawanishi City Hospital, 5-21-1, Kawanishi, Hyogo 666-0195 Japan

**Keywords:** Heart failure with preserved ejection fraction, Controlling nutritional status, Changes in nutritional status, Prognosis

## Abstract

Undernutrition has been identified as a poor prognostic factor in heart failure with preserved ejection fraction (HFpEF). This study aimed to evaluate the impact of changes in nutritional status from discharge to one year post-discharge on the prognosis of patients with HFpEF. Initially, 547 HFpEF cases were classified into a normal nutrition group (NN) (*n* = 130) and an undernutrition group (UN) (*n* = 417), according to Controlling Nutritional Status (CONUT) scores at discharge. These groups were further subdivided according to CONUT scores one year post-discharge into NN (G1, *n* = 88) and UN (G2, *n* = 42), and NN (G3, *n* = 147) and UN (G4, *n* = 270), respectively. The primary end point was defined as a composite of all-cause mortality or heart failure readmission after the visit one year post-discharge. Normal nutrition was defined as a CONUT score of 0 or 1, and undernutrition as a CONUT score of ≥ 2. We compared the incidence rates of the primary end point between G1 and G2, and G3 and G4, and identified predictors for abnormalization or normalization of CONUT score one year post-discharge, as well as covarying factors with change in CONUT. In a multivariable Cox proportional hazards model, abnormalization of CONUT score was associated with an increased risk of the primary end point (adjusted HR [hazard ratio]: 2.87, 95% CI [confidence interval]: 1.32–6.22, *p* = 0.008), while normalization of CONUT was associated with a reduced risk (adjusted HR: 0.40, 95% CI: 0.23–0.67, *p* < 0.001). In a multivariate logistic regression analysis of patients with normal nutrition at discharge, the Euro Qol 5 Dimension score was identified as an independent predictor for abnormalization of CONUT score one year post-discharge (OR: 0.06, 95% CI: 0.01–0.43, *p* = 0.023). Among patients with undernutrition at discharge, prior heart failure hospitalization was the independent predictor for normalization of CONUT score (OR: 0.36, 95% CI: 0.20–0.66, *p* < 0.001). In a multivariate linear regression analysis, independent covariates associated with changes in CONUT included hemoglobin (β = − 0.297, *p* < 0.001), C-reactive protein (β = 0.349, *p* < 0.001), and log NT-proBNP (β = 0.142, *p* < 0.001). Post-discharge abnormalization or normalization of CONUT scores has prognostic impact on patients with HFpEF. Changes in CONUT may independently correlate with changes in hematopoiesis, inflammation, and fluid retention.

## Introduction

HFpEF is a prevalent form of heart failure among elderly patients, with incidence shown to increase with age [1], a trend particularly pronounced in Japan due to its super-aging society. The prognosis of HFpEF is reportedly poor, comparable to that for heart failure with reduced ejection fraction (HFrEF) [2]. Given the likely imminent onset of a heart failure pandemic, the development of effective management strategies for HFpEF has become a critical priority. Currently, sodium–glucose cotransporter-2 (SGLT2) inhibitors are the only agents shown to improve prognosis in patients with HFpEF [3]. Nevertheless, comorbidities are common in HFpEF [4], with malnutrition being particularly significant. Indeed, malnutrition is recognized as a strong prognostic predictor in HFpEF [5–8], and several studies suggest that changes in nutritional status during hospitalization are predictive of post-discharge prognosis [9, 10]. However, the impact of post-discharge changes in nutritional status on the long-term prognosis in patients with HFpEF remains unclear. The CONUT score, developed by de Ulíbari et al. as a screening tool to assess nutritional status in hospital populations [11], utilizes the three parameters—serum albumin, total cholesterol level, and total lymphocyte count—and is widely employed to evaluate nutritional status in heart failure [12, 13]. Here, we investigated the impact of post-discharge changes in nutritional status, as assessed by CONUT score, on long-term prognosis of patients with HFpEF, using clinical data from a multicenter, prospective observational study conducted at Osaka University and its affiliated facilities. Additionally, we examined predictors associated with post-discharge changes in nutritional status. Additionally, we examined predictors associated with post-discharge changes in nutritional status.

## Methods

### The PURSUIT-HFpEF Registry

The PURSUIT-HFpEF study (Prospective Multicenter Observational Study of Patients with Heart Failure with Preserved Ejection Fraction) (UMIN 000021831) is a multicenter, prospective observational study, which enrolled patients hospitalized with decompensated heart failure, as defined by the Framingham criteria. Details of the study have been described previously [14]. Briefly, inclusion criteria required a left ventricular ejection fraction (LV-EF) ≥ 50% on transthoracic echocardiography (TTE) test upon admission and NT-pro BNP ≥ 400 pg/ml or BNP ≥ 100 pg/ml. Exclusion criteria included patients with severe aortic stenosis, aortic regurgitation, mitral stenosis, or mitral regurgitation due to structural valve abnormalities identified by TTE at admission. Patients under 20 years of age, those admitted with acute coronary syndrome, those with a non-cardiac life expectancy of less than six months, recipients of heart transplants, and those deemed unsuitable by the attending physician were also excluded. Thirty-one facilities participated in this study, with no standardized protocol for post-discharge nutritional management. Written informed consent was obtained from all participants, and the study was approved by the ethics committee of each participating institution. This study adheres to the ethical standards set forth by the Declaration of Helsinki, with protocol approval from the institutional review board of all participating facilities.

### Data collection

**Fig. 1:**
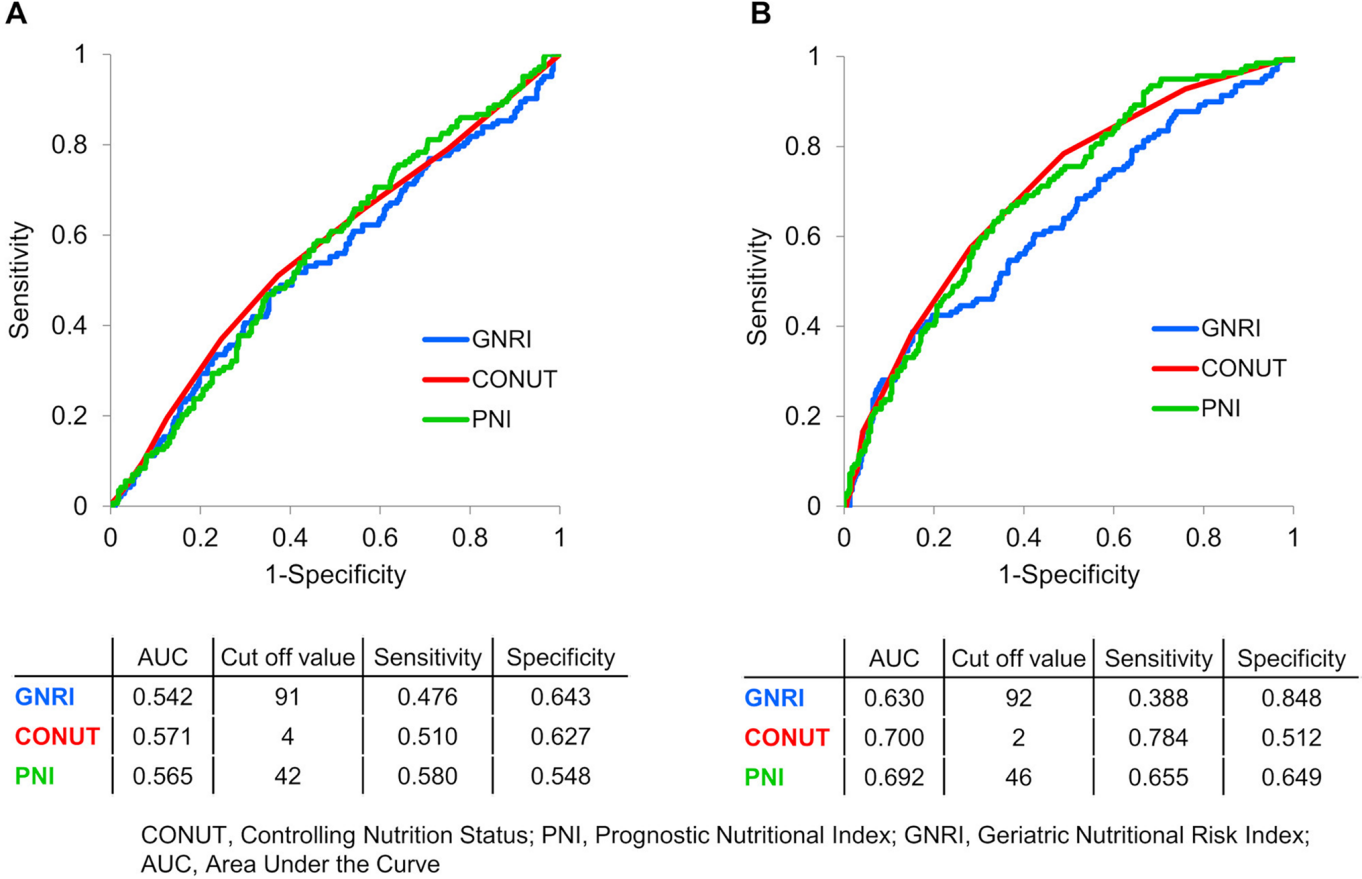
Receiver operating characteristic curve analysis for three nutritional status indices at discharge and one year post-discharge predicting the primary end point. **A** ROC curves at discharge, **B** ROC curves one year post-discharge. *CONUT* Controlling Nutrition Status, *PNI* Prognostic Nutritional Index, *GNRI* Geriatric Nutritional Risk Index, *AUC* area under the curve, *ROC* receiver operating characteristic

We collected comprehensive data including detailed medical history, comorbidities, clinical frailty scale [15], medication history, laboratory and echocardiographic findings, Prognostic Nutritional Index (PNI) [16], Geriatric Nutritional Risk Index (GNRI) [17], and Euro Qol 5 Dimension (EQ-5D) [18]. Each patient was monitored longitudinally, with outcome data collected on all-cause and cardiac mortality, as well as the incidence of heart failure readmissions. Figure 1 presents the receiver operating characteristic (ROC) curve analysis for three nutritional status indices—CONUT, PNI, and GNRI—evaluated at discharge (Fig. 1A) and one year post-discharge (Fig. 1B), predicting the primary end point, a composite of all-cause mortality or heart failure readmission after the visit one year post-discharge. Among the three indices, CONUT demonstrated the highest predictive accuracy for the primary end point both at discharge and one year post-discharge. Therefore, CONUT was selected as the nutritional index for this study. The total lymphocyte number, serum albumin, and total cholesterol levels were measured, and the CONUT score was calculated at both discharge and one year post-discharge using the following formula: CONUT = serum albumin score (≥ 3.5 g/dL [0 point], 3.0–3.4 g/dL [2 points], 2.5–2.9 g/dL [4 points], < 2.5 g/dL [6 points]) + total lymphocyte score (≥ 1600/mL [0 point], 1200–1599/mL [1 point], 800–1199/mL [2 points], < 800/mL [3 points]) + total cholesterol score (≥ 180 mg/dL [0 point], 140–179 mg/dL [1 point], 100–139 mg/dL [2 points], < 100 mg/dL [3 points]). Patient data were recorded by research cardiologists and trained research nurses during hospitalization and subsequently transferred to the data collection center for processing and analysis. Medical history was obtained upon admission, while vital signs, and laboratory and echocardiographic data were documented both at discharge and one year post-discharge.

### Study population

**Fig. 2:**
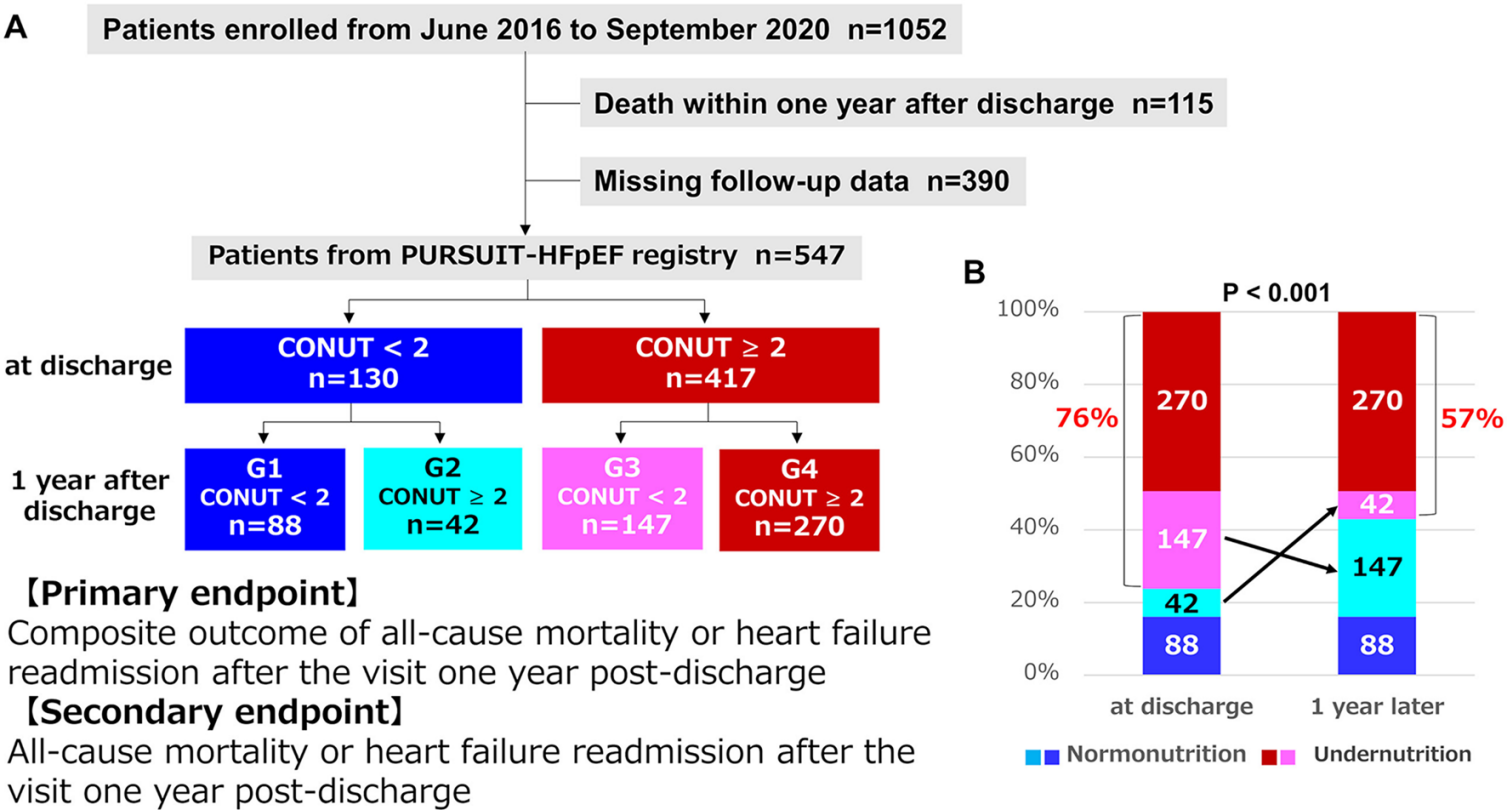
**A** Flowchart illustrating the subgroup classification of the study population and **B** percentage change in undernutrition from discharge to one year post-discharge

This study is a post hoc analysis of the PURSUIT-HFpEF study. Figure 2A presents the flowchart illustrating the subgroup classification of the study population. Of the 1052 patients enrolled in the PURSUIT-HFpEF Registry [14] from June 2016 to September 2020, 390 patients without CONUT data one year post-discharge and 115 patients who died within the first year post-discharge were excluded, leaving a cohort of 547 patients for analysis. Normal nutritional status was defined as a CONUT score of 0 or 1, and undernutrition as a CONUT score ≥ 2, based on the established CONUT criteria [11]. The study cohort (*n* = 547) was classified into four groups (G1–G4) according to nutritional status at discharge and one year post-discharge. Initially, the 547 patients were divided into normal nutrition (*n* = 130) and undernutrition groups (*n* = 417) based on CONUT scores at discharge. Subsequently, the normal nutrition group at discharge was further subdivided into normal nutrition group (G1, *n* = 88) and undernutrition group (G2, *n* = 42) based on CONUT scores one year post-discharge. Similarly, the undernutrition group at discharge was divided into normal nutrition group (G3, *n* = 147) and undernutrition group (G4, *n* = 270) based on the same criteria.

### Clinical outcomes

The primary end point of this study was a composite outcome of all-cause mortality or heart failure readmission after the visit one year post-discharge. Secondary outcomes were all-cause mortality or heart failure admission after that period. Additionally, cardiac death was defined as mortality due to heart failure, myocardial infarction, arrhythmia, sudden death, or other cardiovascular-related causes during the follow-up period.

### Definition of abnormalization or normalization of CONUT

Abnormalization of CONUT was defined as the progression of patients with normal nutritional status (CONUT < 2) at discharge, such as those in groups G1 or G2, to undernutritional status (CONUT ≥ 2) one year post-discharge, similar to those in G2. Conversely, normalization of CONUT was defined as the improvement of patients with undernutrition (CONUT ≥ 2) at discharge, such as in groups G3 or G4, to normal nutritional status (CONUT < 2) one year post-discharge, similar to those in G3.

### Statistical analysis

Continuous variables are reported as median with interquartile range, while categorical data are expressed as percentages. Statistical significance for continuous variables was assessed via the Mann–Whitney *U* test or the Wilcoxon signed-rank test, and for categorical variables, using the Chi-squared test. End points estimation was performed with Kaplan–Meier survival curves, and differences between groups were evaluated using the log-rank test. Hazard ratios (HR) and 95% confidence intervals (CI) for outcomes across subgroups were calculated through a Cox proportional hazards regression model. Factors associated with abnormalization or normalization of CONUT score were analyzed using a logistic regression model, while covariates associated with change in CONUT score were examined through a linear regression model. Univariate regression analyses were first conducted, with statistically significant factors subsequently included in a multivariate regression model. All analyses were performed with bell curve for Excel statistical software (version 4.01, Bell Curve for Excel, Social Survey Research Information Co., Ltd., Tokyo, Japan).

## Results

### The percentage change in undernutrition one year post-discharge

Figure 2B illustrates the percentage change in undernutrition from discharge to one year post-discharge, showing a significant reduction from 76% at discharge to 57% one year post-discharge (*p* < 0.001).

### Patient characteristics at discharge

Table 1 presents the patient characteristics at discharge in the four subgroups, classified by the presence or absence of undernutrition (CONUT ≥ 2) at discharge and one year post- discharge. Among all patients, the median age was 82 [76, 86] years, 47% being male. Heart failure readmission within one year post-discharge occurred in 18% of cases. Among patients with normal CONUT score (< 2) at discharge, those in G2 were significantly older than those in G1, and had lower BMI, total lymphocyte count, total cholesterol, low-density lipoprotein cholesterol, PNI, GNRI, and EQ-5D score. Among patients with abnormal CONUT score (≥ 2) at discharge, those in G3 were significantly younger than those in G4, and had a significantly lower prevalence of chronic kidney disease, prior heart failure hospitalization, prior myocardial infarction, prior percutaneous coronary intervention, and antiplatelet use. Additionally, G3 had significantly higher levels for total lymphocyte count, hemoglobin, eGFR, total cholesterol, low-density lipoprotein cholesterol, triglyceride, and PNI, as well as significantly lower log NT-pro BNP values compared to G4.

**Table 1:** Patient characteristics at discharge

Clinical data	Overall *n* = 547	CONUT at discharge < 2 *n* = 130		*p* value	CONUT at discharge ≥ 2 *n* = 417		*p* value
		G1 n = 88 CONUT 1Y < 2	G2 *n* = 42 CONUT 1Y ≥ 2		G3 *n* = 147 CONUT 1Y < 2	G4 *n* = 270 CONUT 1Y ≥ 2	
Age (years)	82 [76, 86]	79 [74, 84]	83 [78, 86]	0.020	82 [76, 85]	83 [77, 87]	0.024
Male	255 (47%)	36 (41%)	13 (31%)	0.27	65 (44%)	141 (52%)	0.12
Body mass index (kg/m^2^)	24.2 [21.6, 27.2]	25.2 [22.4, 28.3]	23.9 [20.3, 27.1]	0.08	24.3 [22.0, 28.0]	24.1 [21.0, 26.8]	0.12
Clinical frailty scale	3 [2, 4]	3 [2, 4]	3 [2, 5]	0.37	3 [2, 4]	3 [3, 4]	0.15
Hypertension	471 (86%)	73 (83%)	30 (71%)	0.13	130 (88%)	238 (88%)	0.93
Atrial fibrillation	250 (46%)	39 (44%)	21 (50%)	0.58	68 (46%)	122 (45%)	0.84
Diabetes	188 (34%)	29 (33%)	17 (40%)	0.40	46 (31%)	96 (36%)	0.38
Dyslipidemia	244 (45%)	35 (40%)	14 (33%)	0.48	71 (48%)	124 (46%)	0.64
CKD history	214 (39%)	21 (24%)	13 (31%)	0.39	50 (34%)	130 (48%)	0.005
COPD	40 (7%)	4 (5%)	0 (0%)	0.16	13 (9%)	23 (9%)	0.91
Smoking history	211 (39%)	28 (32%)	13 (31%)	0.92	53 (36%)	117 (43%)	0.15
Prior HF hospitalization	131 (24%)	21 (24%)	9 (21%)	0.76	18 (12%)	83 (31%)	< 0.001
HFR within one year	96 (18%)	9 (10%)	9 (21%)	0.08	23 (16%)	55 (20%)	0.24
NYHAI	216 (39%)	38 (43%)	23 (55%)	0.22	56 (38%)	99 (37%)	0.77
NYHAII	305 (56%)	48 (55%)	18 (43%)	0.21	84 (57%)	155 (57%)	0.96
NYHAIII or IV	26 (5%)	2 (2%)	1 (2%)	0.97	7 (5%)	16 (6%)	0.62
Prior myocardial infarction	39 (7%)	0 (0%)	1 (2%)	0.15	4 (3%)	34 (13%)	< 0.001
Prior PCI	71 (13%)	4 (5%)	4 (10%)	0.27	13 (9%)	50 (19%)	0.008
Prior stroke	74 (14%)	6 (7%)	4 (10%)	0.59	27 (18%)	37 (14%)	0.21
Systolic BP (mmHg)	119 [106, 132]	117 [106, 130]	118 [110, 128]	0.81	122 [112, 133]	119 [105, 133]	0.19
Heart rate (b.p.m.)	68 [60, 77]	70 [61, 77]	67 [62, 76]	0.38	69 [61, 78]	68 [60, 76]	0.21
Echocardiography							
LVEF (%)	64 [59, 69]	64 [60, 70]	63 [56, 68]	0.24	65 [59, 69]	64 [59, 70]	0.76
LAD (mm)	44 [39, 49]	45 [39, 51]	45 [40, 52]	0.47	43 [39, 47]	44 [39, 49]	0.08
Average E/e'	12.2 [9.6, 16.3]	11.2 [8.9, 14.7]	11.7 [10.1, 15.3]	0.33	12.5 [9.6, 16.5]	12.7 [9.5, 16.4]	0.96
TRPG (mmHg)	26 [22, 32]	25 [21, 31]	28 [24, 33]	0.17	25 [21, 32]	27 [23, 33]	0.028
Laboratory data							
Total lymphocytes (/μL)	1400 [1104, 1782]	1938 [1699, 2213]	1716 [1603, 1899]	< 0.001	1472 [1163, 1767]	1172 [923, 1440]	< 0.001
Hemoglobin (g/dL)	11.4 [10.3, 12.9]	13.0 [11.7, 14.2]	12.4 [11.3, 13.9]	0.32	11.1 [10.1, 12.9]	10.9 [9.7, 12.1]	0.014
Albumin (g/dL)	3.5 [3.2, 3.8]	3.8 [3.7, 4.0]	3.7 [3.6, 4.0]	0.25	3.3 [3.1, 3.6]	3.4 [3.1, 3.7]	0.43
BUN (mg/dL)	24 [18, 33]	21 [17, 29]	25 [16, 32]	0.47	22 [17, 31]	26 [19, 37]	0.001
eGFR (mL/min/1.73 m^2^)	44 [32, 55]	48 [39, 61]	45 [34, 51]	0.08	45 [35, 59]	40 [25, 51]	< 0.001
Total cholesterol (mg/dL)	160 [137, 184]	184 [161, 210]	170 [156, 188]	0.048	159 [140, 182]	150 [125, 171]	0.003
LDL cholesterol (mg/dL)	**93 [74, 112]**	**109 [94, 133]**	**101 [91, 114]**	**0.011**	**92 [77, 110]**	**84 [66, 105]**	**0.005**
HDL cholesterol (mg/dL)	**43 [36, 52]**	**46 [38, 54]**	**46 [39, 59]**	**0.58**	**43 [36, 51]**	**42 [34, 50]**	**0.61**
Triglyceride	**99 [75, 130]**	**115 [85, 154]**	**106 [92, 134]**	**0.36**	**103 [80, 131]**	**92 [69, 120]**	**0.008**
HbA1c (%)	6.0 [5.6, 6.6]	6.1 [5.8, 6.5]	6.1 [5.6, 6.7]	0.90	6.0 [5.6, 6.5]	5.9 [5.6, 6.6]	0.99
CRP (mg/dL)	0.2 [0.1, 0.6]	0.2 [0.1, 0.4]	0.1 [0.1, 0.2]	0.27	0.3 [0.1, 0.7]	0.3 [0.1, 0.8]	0.88
Log NT-proBNP	6.8 [6.0, 7.5]	6.4 [5.5, 7.1]	6.6 [5.7, 7.2]	0.25	6.7 [6.0, 7.4]	7.1 [6.2, 7.9]	0.006
Scoring system							
CONUT score	3 [2, 5]	1 [0, 1]	1 [1]	0.007	3 [2, 5]	4 [3, 5]	< 0.001
PNI score	42 [38, 46]	48 [46, 51]	46 [44, 49]	0.003	42 [38, 44]	40 [37, 43]	0.004
GNRI score	94 [87, 101]	102 [96, 108]	96 [90, 105]	0.013	93 [85, 99]	92 [85, 98]	0.53
EQ-5D score	0.80 [0.65, 0.90]	0.85 [071, 1.00]	0.78 [0.56, 0.87]	0.005	0.83 [0.63, 0.97]	0.78 [0.64, 0.90]	0.48
Medication							
ACEi/ARB	312 (57%)	51 (58%)	20 (48%)	0.27	87 (59%)	154 (57%)	0.67
Beta-blocker	314 (57%)	46 (52%)	28 (67%)	0.12	80 (54%)	160 (59%)	0.34
Aldosterone antagonist	209 (38%)	35 (40%)	16 (38%)	0.85	60 (41%)	98 (36%)	0.36
SGLT2i	35 (6%)	9 (10%)	3 (7%)	0.57	4 (3%)	19 (7%)	0.07
Diuretics	446 (82%)	66 (75%)	34 (81%)	0.51	119 (81%)	227 (84%)	0.42
Statin	191 (35%)	19 (22%)	11 (26%)	0.56	58 (39%)	103 (38%)	0.79
Antiplatelet	171 (31%)	19 (22%)	7 (17%)	0.64	37 (25%)	108 (40%)	0.003
Anticoagulant	336 (61%)	55 (63%)	33 (79%)	0.07	86 (59%)	162 (60%)	0.83

Continuous variables are expressed as median [interquartile range]

CKD chronic kidney disease, COPD chronic obstructive pulmonary disease, HF heart failure, HFR heart failure readmission, NYHA New York Heart Association, PCI percutaneous coronary intervention, BP blood pressure, LV-EF left ventricular ejection fraction, LAD left atrial diameter, TRPG tricuspid regurgitation pressure gradient, BUN blood urea nitrogen, eGFR estimated glomerular filtration rate, LDL low-density lipoprotein, HDL high-density lipoprotein, CRP C-reactive protein, NT-proBNP N-terminal pro-brain natriuretic peptide, CONUT Controlling Nutrition Status, PNI Prognostic Nutritional Index, GNRI Geriatric Nutritional Risk Index, EQ-5D Euro Qol 5 Dimension, ACEi angiotensin-converting-enzyme inhibitor, ARB angiotensin II receptor blocker, SGLT2i sodium/glucose cotransporter 2 inhibitor

### Outcomes

**Fig. 3:**
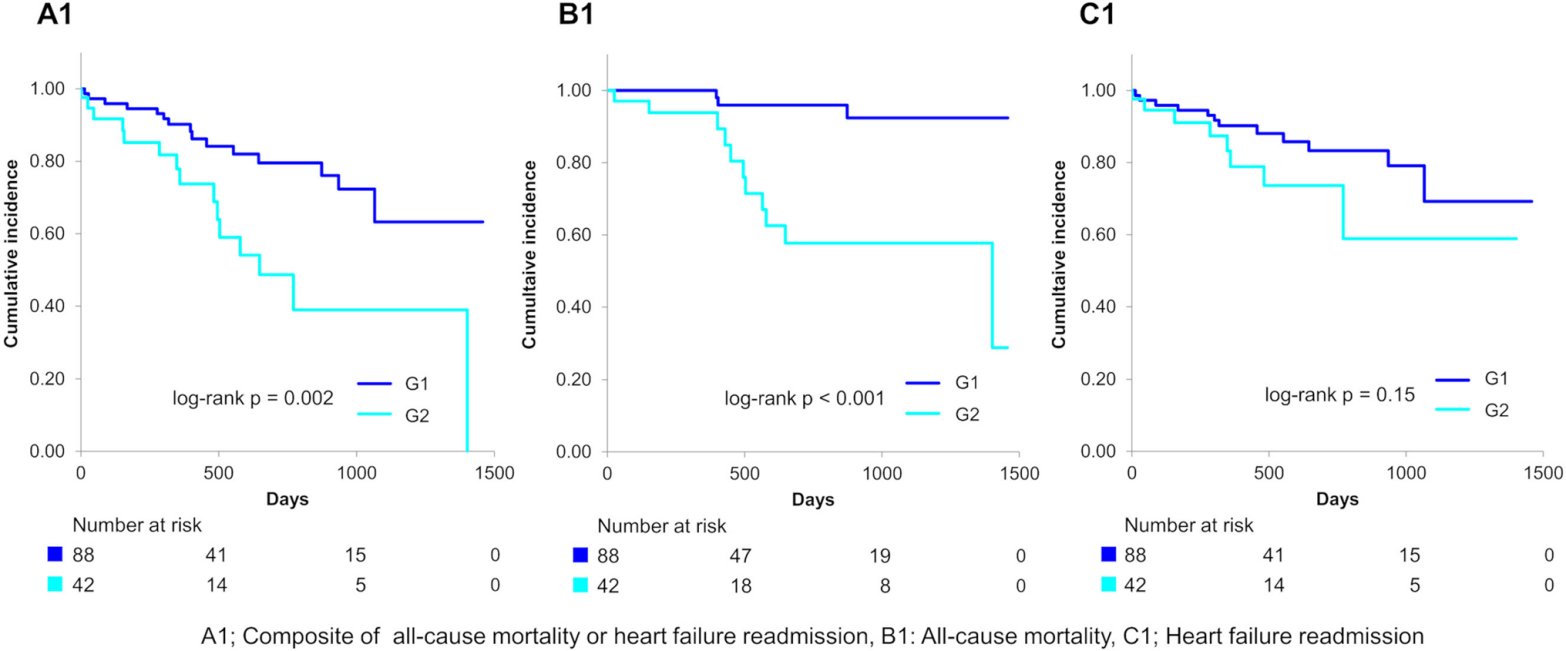
Kaplan–Meier curves and log-rank test after the visit one year post-discharge for G1 and G2. A1 Composite of all-cause mortality or heart failure readmission, B1 all-cause mortality, C1 heart failure readmission

**Fig. 4:**
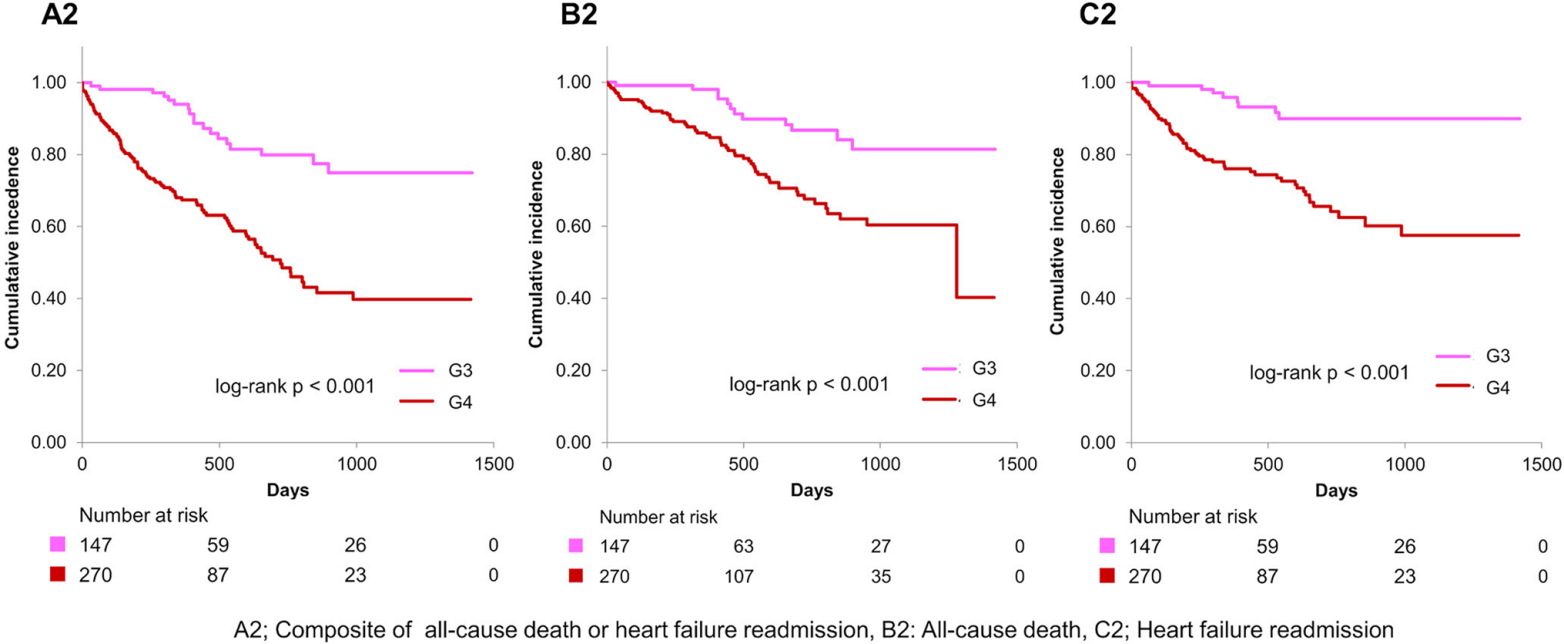
Kaplan–Meier curves and log-rank test after the visit one year post-discharge for G3 and G4. A2 Composite of all-cause mortality or heart failure readmission, B2 all-cause mortality, C2 heart failure readmission

**Fig. 5:**
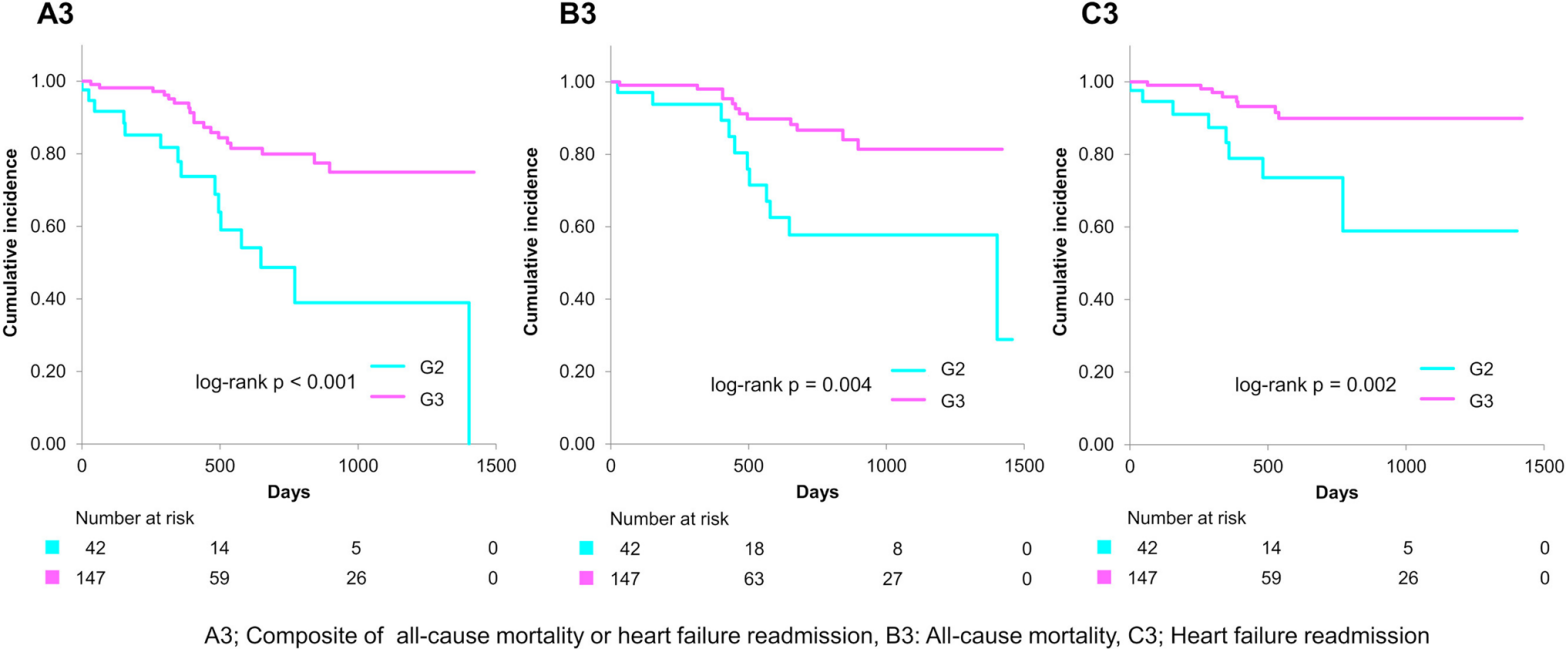
Kaplan–Meier curves and log-rank test after the visit one year post-discharge for G2 and G3. A3 Composite of all-cause mortality or heart failure readmission, B3 all-cause mortality, C3 heart failure readmission

**Fig. 6:**
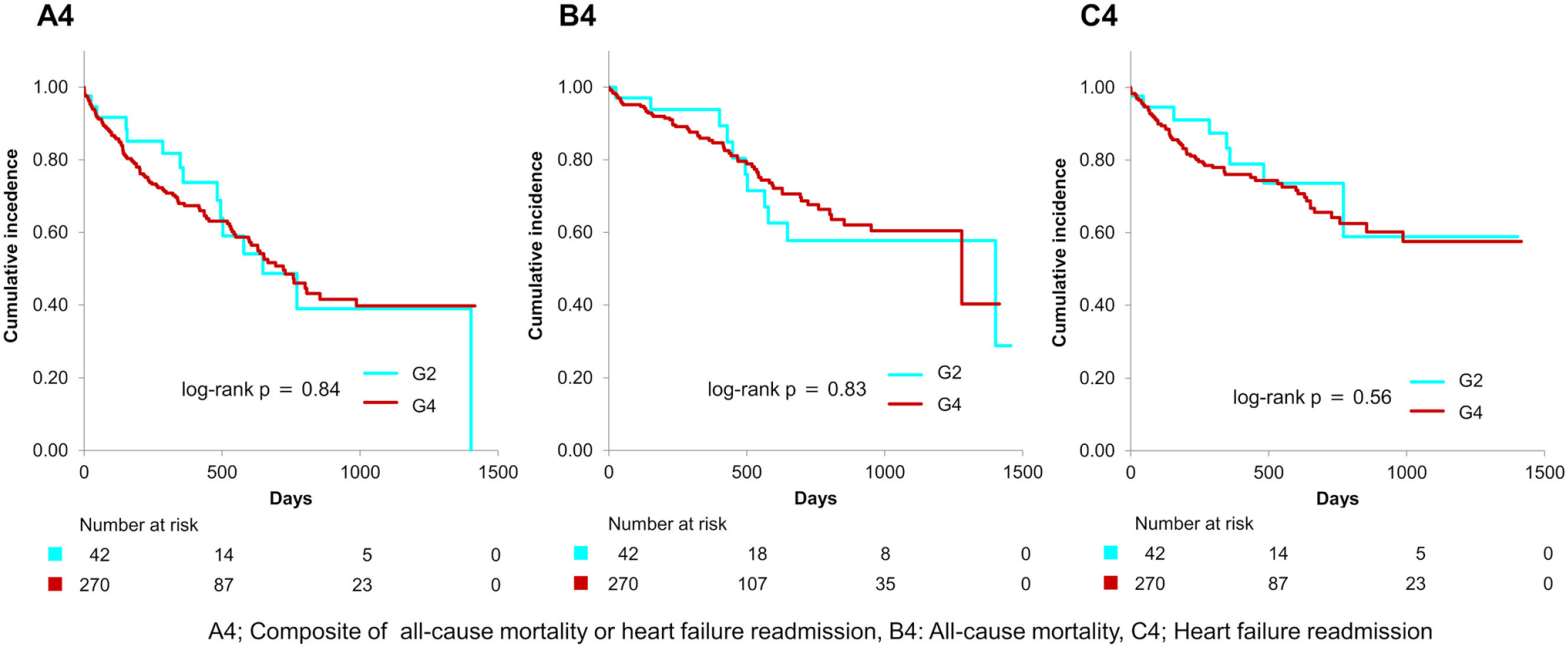
Kaplan–Meier curves and log-rank test after the visit one year post-discharge for G2 and G4. A4 Composite of all-cause mortality or heart failure readmission, B4 all-cause mortality, C4 heart failure readmission

**Fig. 7:**
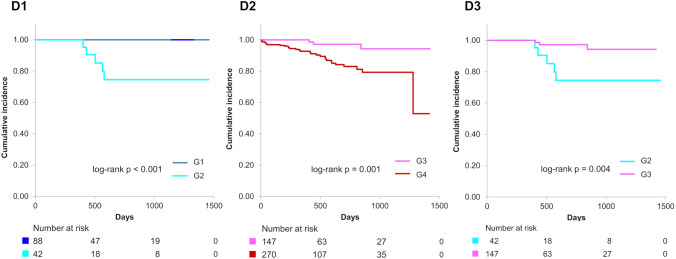
Kaplan–Meier curves and log-rank test for cardiac mortality after the visit one year post-discharge between each pair of subgroups

Figure 3 illustrates that primary end point and all-cause mortality were significantly elevated in G2 compared to G1 (log-rank *p* = 0.002 for the primary end point and < 0.001 for all-cause mortality) over a mean follow-up duration of 482 ± 387 days. Figure 4 demonstrates that the primary end point, all-cause mortality, and heart failure readmission were significantly higher in G4 than in G3 (log-rank *p* < 0.001 for each of the primary end point, all-cause mortality, and heart failure readmission). Notably, the primary end point, all-cause mortality, and heart failure readmission in G2 were considerably higher than in G3 (log-rank *p* < 0.001 for the primary end point, *p* = 0.004 for all-cause mortality, and *p* = 0.002 for heart failure readmission) (Fig. 5). Additionally, the primary end point, all-cause mortality, and heart failure readmission in G2 were comparable to that in G4 (log-rank *p* = 0.84 for the primary end point, *p* = 0.83 for all-cause mortality, and *p* = 0.56 for heart failure readmission) (Fig. 6). Tables 2 and 3 present the results of the multivariate Cox proportional hazards analysis for each end point among patients with normal or abnormal CONUT scores at discharge. Abnormalization of CONUT score one year post-discharge was associated with an elevated risk of the primary end point (adjusted HR [hazard ratio]: 2.55, 95% CI [confidence interval], 1.13–5.77, *p* = 0.025) among patients with normal CONUT scores at discharge (Table 2). Conversely, normalization of CONUT score one year post-discharge was associated with a reduced risk (adjusted HR: 0.40, 95% CI, 0.23–0.67, *p* < 0.001) among patients with abnormal CONUT score at discharge (Table 3). Additionally, a total of 36 cardiac deaths were documented, comprising 21 cases of heart failure, 1 case of myocardial infarction, 4 cases of arrhythmia or sudden cardiac death, and 10 attributed to other causes. Kaplan–Meier survival analysis indicated that the incidence of cardiac mortality was significantly higher in Group 2 than in Group 1 (log-rank *p* < 0.001), significantly lower in Group 3 than in Group 4 (log-rank *p* = 0.001), and also significantly lower in Group 3 compared to Group 2 (log-rank *p* = 0.004) (Fig. 7).

**Table 2:** Hazard risk associated with the abnormalization of CONUT score for each end point in patients with normal CONUT scores at discharge

	Subgroup	No. of patients	No. of patients with event (%)	Unadjusted		Adjusted*	
				HR (95% CI)	*p* value	HR (95% CI)	*p* value
Primary end point	**G1**	88	15 (17)	1.00		1.00	
	**G2**	42	15 (36)	2.99 (1.45–6.16)	0.003	**2.55** (**1.13**–**5.77**)	**0.025**
All-cause mortality	**G1**	88	3 (3)	1.00		1.00	
	**G2**	42	11 (26)	9.35 (2.60–33.6)	< 0.001	**8.24** (**2.22**–**30.6**)	**0.002**
Heart failure readmission	**G1**	88	12 (14)	1.00		1.00	
	**G2**	42	8 (19)	1.94 (0.78–4.78)	0.15	**1.67** (**0.65**–**4.26**)	**0.29**

No. number, HR hazard ratio, CI confidence interval

*The primary end point was adjusted for the clinical frailty scale and the Euro Qol 5 Dimension. All-cause mortality was adjusted for body mass index. Heart failure readmission was adjusted for heart failure readmission within one year post-discharge

**Table 3:** Hazard risk associated with the normalization of CONUT score for each end point in patients with abnormal CONUT scores at discharge

	Subgroup	No. of patients	No. of patients with event (%)	Unadjusted		Adjusted*	
				HR (95% CI)	*p* value	HR (95% CI)	*p* value
Primary end point	**G3**	147	18 (12)	0.28 (0.17–0.46)	< 0.001	**0.40** (**0.23**–**0.67**)	< 0.001
	**G4**	270	100 (37)	1.00		1.00	
All-cause mortality	**G3**	147	12 (8)	0.34 (0.18–0.63)	< 0.001	**0.56** (**0.29**–**1.07**)	**0.08**
	**G4**	270	60 (22)	1.00		1.00	
Heart failure readmission	**G3**	147	8 (5)	0.20 (0.10–0.41)	< 0.001	**0.27** (**0.13**–**0.58**)	< 0.001
	**G4**	270	64 (24)	1.00		1.00	

No. number, HR hazard ratio, CI confidence interval

*The primary end point was adjusted for age, clinical frailty scale, prior heart failure hospitalization, heart failure readmission within one year post-discharge, estimated glomerular filtration rate, log N-terminal pro-brain natriuretic peptide, and CONUT score at discharge. All-cause mortality was adjusted for age, clinical frailty scale, prior heart failure hospitalization, heart failure readmission within one year post-discharge, log N-terminal pro-brain natriuretic peptide, and CONUT score at discharge. Heart failure readmission was adjusted for age, prior heart failure hospitalization, estimated glomerular filtration rate, and log N-terminal pro-brain natriuretic peptide

### Factors associated with the abnormalization or normalization of CONUT score

Multivariate logistic regression analysis revealed that EQ-5D score (OR: 0.06, 95% CI, 0.01–0.43, *p* = 0.006) were an independent factor associated with the abnormalization of CONUT score one year post-discharge among patients with normal CONUT score at discharge (Table 4). Conversely, prior heart failure hospitalization (OR: 0.36, 95% CI, 0.20–0.66, p < 0.001) was an independent factor associated with the normalization of CONUT score one year post-discharge among patients with abnormal CONUT score at discharge (Table 5).

**Table 4:** Factors associated with the abnormalization of CONUT score in patients with normal CONUT scores at discharge

Variable	Univariate analysis			Multivariate analysis		
	OR	95% CI	*p* value	OR	95% CI	*p* value
Age	1.05	1.00–1.10	0.039	**1.04**	**0.99–1.09**	**0.09**
Male	0.65	0.30–1.41	0.27			
Body mass index	0.93	0.85–1.01	0.10			
Clinical frailty scale	1.10	0.89–1.36	0.36			
Atrial fibrillation	1.26	0.60–2.62	0.54			
Diabetes	1.38	0.65–2.96	0.40			
COPD	–	–	–			
Prior HF hospitalization	0.87	0.36–2.11	0.76			
HFR within one year after discharge	2.39	0.87–6.57	0.09			
Systolic blood pressure	1.00	0.98–1.03	0.76			
Heart rate	0.99	0.96–1.02	0.44			
Hemoglobin	0.92	0.75–1.13	0.43			
eGFR	0.98	0.95–1.00	0.07			
CRP	0.69	0.25–1.90	0.47			
Log NT-proBNP	1.31	0.89–1.92	0.18			
EQ-5D score	0.05	0.01–0.41	0.005	**0.06**	**0.01–0.43**	**0.006**
ACEi/ARB	0.66	0.32–1.38	0.27			
Aldosterone antagonist	0.93	0.44–1.98	0.85			
SGLT2i	0.68	0.17–2.64	0.57			
Statin	1.29	0.55–3.03	0.56			

OR odds ratio, CI confidence interval

COPD chronic obstructive pulmonary disease, HF heart failure, HFR HF readmission, eGFR estimated glomerular filtration rate, NT-proBNP N-terminal pro-brain natriuretic peptide, EQ-5D Euro Qol 5 Dimension, ACEi angiotensin-converting-enzyme inhibitor, ARB angiotensin II receptor blocker, SGLT2i sodium/glucose cotransporter 2 inhibitor

**Table 5:** Factors associated with the normalization of CONUT score in patients with abnormal CONUT scores at discharge

Variable	Univariate analysis			Multivariate analysis		
	OR	95% CI	*p* value	OR	95% CI	*p* value
Age	0.97	0.95–0.99	0.014	**0.98**	**0.95–1.00**	**0.06**
Male	0.73	0.48–1.09	0.12			
Body mass index	1.04	1.00–1.09	0.058			
Clinical frailty scale	0.91	0.79–1.04	0.15			
Atrial fibrillation	1.04	0.70–1.56	0.83			
Diabetes	0.83	0.54–1.28	0.41			
COPD	1.04	0.51–2.12	0.91			
Prior HF hospitalization	0.31	0.18–0.55	< 0.001	**0.36**	**0.20–0.66**	**< 0.001**
HFR within one year after discharge	0.73	0.42–1.24	0.24			
Systolic blood pressure	1.01	1.00–1.02	0.20			
Heart rate	1.01	0.99–1.03	0.22			
Hemoglobin	1.16	1.04–1.29	0.010	**1.05**	**0.93–1.28**	**0.45**
eGFR	1.02	1.01–1.03	< 0.001	**1.01**	**1.00–1.02**	**0.18**
C-reactive protein	1.05	0.91–1.20	0.52			
Log NT-proBNP	0.78	0.65–0.93	0.007	**0.87**	**0.70–1.07**	**0.18**
EQ-5D score	1.03	0.36–2.98	0.96			
ACEi/ARB	1.09	0.73–1.64	0.67			
Aldosterone antagonist	1.21	0.80–1.83	0.36			
SGLT2i	0.37	0.12–1.11	0.08			
Statin	1.06	0.70–1.60	0.79			

OR odds ratio, CI confidence interval

COPD chronic obstructive pulmonary disease, HF heart failure, HFR HF readmission, eGFR estimated glomerular filtration rate, NT-proBNP N-terminal pro-brain natriuretic peptide, EQ-5D Euro Qol 5 Dimension, ACEi angiotensin-converting-enzyme inhibitor, ARB angiotensin II receptor blocker, SGLT2i sodium/glucose cotransporter 2 inhibitor

### Covariates associated with changes in CONUT score one year post- discharge

In all patients, multivariate linear regression analysis revealed that changes in hemoglobin (β = − 0.297, *p* < 0.001), C-reactive protein (β = 0.349, *p* < 0.001), and log NT-proBNP (β = 0.142, *p* < 0.001) were independently associated with changes in CONUT scores (Table 6).

**Table 6:** Covarying factors associated with changes in CONUT from discharge to one year after discharge

Variables	Univariate analysis		Multivariate analysis	
	β	p value	β	p value
⊿ Body mass index	− 0.007	0.87	0.012	0.77
⊿ Systolic blood pressure	0.033	0.44	0.023	0.57
⊿ Heart rate	− 0.023	0.60	− 0.027	0.51
⊿ Hemoglobin	− 0.413	< 0.001	− 0.297	< 0.001
⊿ eGFR	0.092	0.032	0.071	0.082
⊿ Log NT-proBNP	0.232	< 0.001	0.142	< 0.001
⊿ C-reactive protein	0.384	< 0.001	0.349	< 0.001

eGFR estimated glomerular filtration rate, NT-proBNP N-terminal pro-brain natriuretic peptide

## Discussion

### Main findings

In this post hoc analysis of a prospective observational study, we have demonstrated the following key points.

1. In the group with normal nutrition, as evaluated by CONUT at discharge, the abnormalization of nutritional status one year post-discharge was significantly associated with a poor prognosis.
2. In the group with undernutrition, as assessed by CONUT at discharge, the normalization of nutritional status one year post-discharge was significantly associated with a better prognosis.
3. In the group with normal CONUT score at discharge, lower EQ-5D score was associated with the abnormalization of CONUT one year post-discharge.
4. In the group with abnormal CONUT score at discharge, the absence of prior heart failure hospitalization was associated with the normalization of CONUT one year post-discharge.
5. Changes in nutritional status independently correlated with changes in hemoglobin, C-reactive protein, and log NT-proBNP.

This is the first to underscore the significance of long-term changes in nutritional status, evaluated by CONUT, among patients with HFpEF, highlighting the need for continuous nutritional assessment to enhance patient outcomes.

### Significance of the current study and comparison with previous studies

Our study demonstrated that the prognosis of patients whose CONUT became abnormal one year post-discharge (G2) was significantly worse than that of those with persistent normal CONUT (G1) (Fig. 3). Moreover, the prognosis of patients whose CONUT normalized one year post-discharge (G3) was significantly better than that of those with persistent abnormal CONUT (G4) (Fig. 4). Several studies have examined the association between changes in nutritional status during hospitalization and prognosis [9, 10, 19]. In particular, the prognosis of patients whose nutritional status normalized, as assessed by CONUT during hospitalization, was significantly better than that of those with persistent undernutrition [10]. Our current findings align with this observation and further demonstrate that normalization of nutritional status after discharge is also associated with a better prognosis in patients with HFpEF. In contrast, another study from our group, which classified patients based on nutritional status at admission and discharge, revealed that a deterioration in nutritional status was associated with a worse long-term prognosis only in patients with undernutrition at admission, but not in those without undernutrition [19]. This discrepancy suggests that the effect of baseline nutritional status on the relationship between nutritional change and subsequent prognosis may vary between the acute and chronic phases. Additionally, our study found that the prognosis of patients with CONUT normalization (G3) was significantly better than that of those with CONUT abnormalization (G2) (Fig. 5). Furthermore, the prognosis of patients with CONUT abnormalization (G2) was comparable to that of those in persistent abnormal CONUT (G4) (Fig. 6). To our knowledge, no previous study has made such comparisons. These findings suggest that maintaining of normal nutritional status or achieving normalization of undernutrition for an extended period post-discharge may be critical for improving prognosis in patients with HFpEF.

### Factors associated with the abnormalization or normalization of nutritional status

In the group with normal nutrition at discharge, lower EQ-5D score was associated with the abnormalization of nutritional status one year post-discharge (Table 4). Health-related QOL, as measured by EQ-5D, was significantly lower in older adults at high risk of malnutrition [20], which is consistent with the results of this study. A significant association between physical frailty, nutritional status, and QOL has been reported in a study of older adults with cardiovascular disease [21], suggesting that it may be necessary to comprehensively evaluate physical frailty and QOL in addition to nutritional status. In the group with abnormal nutrition at discharge, the absence of prior heart failure hospitalization was associated with the normalization of nutritional status one year post-discharge (Table 5). Previous studies have indicated that patients with a history of hospitalization for heart failure exhibit significantly higher rates of comorbidities, including diabetes, atrial fibrillation, and prior myocardial infarction, compared to those without such a history [22, 23]. They also have markedly lower hemoglobin levels, reduced renal function, and significantly elevated BNP levels [23, 24]. Reduced renal function and elevated BNP are associated with fluid overload. In patients with heart failure, fluid overload may induce intestinal edema and impair nutrient absorption [25], thereby hindering efforts to improve malnutrition.

### The relationship between change in nutritional status and hematopoiesis, inflammation, and fluid retention

Changes in CONUT scores were correlated with variations in hemoglobin, C-reactive protein, and log NT-proBNP (Table 6). Hemoglobin levels are significantly associated with nutritional status in patients with heart failure [26]. The CONUT score has been reported to exhibit a significant positive correlation with CRP and a significant negative correlation with hemoglobin in patients with chronic heart failure [27]. The two studies align with and support the findings of our research. Among hospitalized patients with heart failure, those identified as being malnourished or at risk of malnutrition, as assessed by the Mini Nutritional Assessment, tend to exhibit elevated NT-proBNP levels [28], thereby reinforcing our findings. Iron deficiency, the predominant etiology of anemia among patients with heart failure [29], is closely associated with factors including malnutrition, fluid overload, and chronic inflammation [30]. These interconnected disruptions in nutritional status can deteriorate the clinical outcomes of heart failure, warranting comprehensive and multidisciplinary therapeutic interventions.

### Evidence of nutritional support and intervention

A meta-analysis demonstrated that nutritional support and interventions for hospitalized patients with malnutrition are significantly associated with improvements in key clinical outcomes [31]. A recent study highlighted that short-term, individualized in-hospital nutritional support enhanced 30-day outcomes for patients hospitalized with heart failure [32]. A multicenter, randomized, controlled trial reported that tailored nutritional intervention for hospitalized patients with heart failure and malnutrition significantly reduced the risk of all-cause mortality and heart failure readmissions [33]. These findings suggest that inpatient nutritional intervention could play a pivotal role in enhancing the prognosis of malnourished patients with heart failure during hospitalization. However, the effectiveness of long-term nutritional interventions for outpatients with heart failure remains unexamined, and relevant data are currently lacking. A multicenter, randomized, controlled trial investigating specific nutritional supplementation methods found that high-protein oral nutritional supplements reduced mortality and improved the metrics of nutritional status in hospitalized, malnourished older adults compared with placebo [34]. Nonetheless, no high-quality clinical trial with robust evidence has been conducted to evaluate the efficacy of dietary interventions and nutritional supplements in patients with heart failure. Large-scale, randomized controlled trials are necessary to comprehensively establish the clinical efficacy of these interventions [35]. Currently, the development of specific intervention strategies for malnourished patients remains an area of ongoing investigation.

### Limitations

This study is subject to several limitations. First, the study population includes only patients with blood test data available one year post-discharge. Patients who died within one year post-discharge were excluded, which may have resulted in an underestimation of events during this period. Such exclusions inevitably introduce biases. Second, the absence of data on nutritional management and exercise therapy following discharge precludes the evaluation of their influence on the outcomes. Third, the study predominantly examines elderly patients with HFpEF in Japan, limiting the generalizability of the findings to HFpEF populations in other regions. Fourth, the effect of changes in nutritional status on the prognosis of patients with persistent undernutrition remains unverified. Large-scale prospective studies integrating standardized pharmacological, nutritional, and exercise therapy are necessary to validate the impact of post-discharge changes in nutritional status on the prognosis of patients with HFpEF.

## Conclusion

Post-discharge changes in nutritional status significantly influence the prognosis of patients with HFpEF. Maintaining normal nutritional status or correcting undernutrition post-discharge is essential for improving the prognosis of patients with HFpEF, whereas a deterioration in nutritional status is associated with poorer outcomes. This study highlights the critical importance of routine nutritional status assessment in clinical practice for patients with HFpEF, particularly in the presence of significant changes in hemoglobin, C-reactive protein, and NT-proBNP levels.

## Data Availability

The data that support the findings of this study are not publicly available.

## Appendix

The OCVC-Heart Failure Investigators

Masahiro Seo, Tetsuya Watanabe, and Takahisa Yamada, Osaka General Medical Center, Osaka, Japan; Takaharu Hayashi and Yoshiharu Higuchi, Osaka Police Hospital, Osaka, Japan; Masaharu Masuda, Mitsutoshi Asai, and Toshiaki Mano, Kansai Rosai Hospital, Amagasaki, Japan; Hisakazu Fuji, Kobe Ekisaikai Hospital, Kobe, Japan; Daisaku Masuda, Shunsuke Tamaki, Ryu Shutta, and Shizuya Yamashita, Rinku General Medical Center, Izumisano, Japan; Masami Sairyo and Yusuke Nakagawa, Kawanishi City Hospital, Kawanishi, Japan; Haruhiko Abe, Yasunori Ueda, and Yasushi Matsumura, National Hospital Organization Osaka National Hospital, Osaka, Japan; Kunihiko Nagai, Ikeda Municipal Hospital, Ikeda, Japan; Masamichi Yano, Masami Nishino, and Jun Tanouchi, Osaka Rosai Hospital, Sakai, Japan; Yoh Arita and, Nobuyuki Ogasawara, Japan Community Health Care Organization Osaka Hospital, Osaka, Japan; Takamaru Ishizu, Minoru Ichikawa and Yuzuru Takano, Higashiosaka City Medical Center, Higashiosaka, Japan; Eisai Rin, Kawachi General Hospital, Higashiosaka, Japan; Yukinori Shinoda, Koichi Tachibana and Shiro Hoshida, Yao Municipal Hospital, Yao, Japan; Masahiro Izumi, Kinki Central Hospital, Itami, Japan; Hiroyoshi Yamamoto and Hiroyasu Kato, Japan Community Health Care Organization, Osaka Minato Central Hospital, Osaka, Japan; Kazuhiro Nakatani and Yuji Yasuga, Sumitomo Hospital, Osaka, Japan; Mayu Nishio and Keiji Hirooka, Saiseikai Senri Hospital, Suita, Japan; Takahiro Yoshimura and Yoshinori Yasuoka, National Hospital Organization Osaka Minami Medical Center, Kawachinagano, Japan; Akihiro Tani, Kano General Hospital, Osaka, Japan; Yasushi Okumoto, Kinan Hospital, Tanabe, Japan; Yasunaka Makino, Hyogo Prefectural Nishinomiya Hospital, Nishinomiya, Japan; Toshinari Onishi and Katsuomi Iwakura, Sakurabashi Watanabe Hospital, Osaka, Japan; Yoshiyuki Kijima, Japan Community Health Care Organization, Hoshigaoka Medical Center, Hirakata, Japan; Takashi Kitao and Hideyuki Kanai, Minoh City Hospital, Minoh, Japan; Masashi Fujita, Osaka International Cancer Institute, Osaka, Japan; Koichiro Harada, Suita Municipal Hospital, Suita, Japan; Masahiro Kumada and Osamu Nakagawa, Toyonaka Municipal Hospital, Toyonaka, Japan; Ryo Araki and Takayuki Yamada, Otemae Hospital, Osaka, Japan; Akito Nakagawa and Yoshio Yasumura, Amagasaki Chuo Hospital, Amagasaki, Japan; and Taiki Sato, Akihiro Sunaga, Bolrathanak Oeun, Hirota Kida, Yohei Sotomi, Tomoharu Dohi, Kei Nakamoto, Katsuki Okada, Fusako Sera, Hidetaka Kioka, Tomohito Ohtani, Toshihiro Takeda, Daisaku Nakatani, Hiroya Mizuno, Shungo Hikoso, and Yasushi Sakata, Osaka University Graduate School of Medicine, Suita, Japan.
